# Genomic epidemiology of antifungal resistance in human and avian isolates of *Candida albicans*: a pilot study from the One Health perspective

**DOI:** 10.3389/fvets.2024.1345877

**Published:** 2024-02-16

**Authors:** Marianna Domán, Eszter Kaszab, Levente Laczkó, Krisztina Bali, László Makrai, Renátó Kovács, László Majoros, Krisztián Bányai

**Affiliations:** ^1^HUN-REN Veterinary Medical Research Institute, Budapest, Hungary; ^2^National Laboratory for Infectious Animal Diseases, Antimicrobial Resistance, Veterinary Public Health and Food Chain Safety, Budapest, Hungary; ^3^One Health Institute, University of Debrecen, Debrecen, Hungary; ^4^HUN-REN-UD Conservation Biology Research Group, University of Debrecen, Debrecen, Hungary; ^5^Autovakcina Ltd., Budapest, Hungary; ^6^Department of Medical Microbiology, Faculty of Medicine, University of Debrecen, Debrecen, Hungary; ^7^Department of Pharmacology and Toxicology, University of Veterinary Medicine, Budapest, Hungary

**Keywords:** yeast, aneuploidy, loss of heterozygosity, drug resistance, whole genome sequencing

## Abstract

Stress-induced genomic changes in *Candida albicans* contribute to the adaptation of this species to various environmental conditions. Variations of the genome composition of animal-origin *C. albicans* strains are largely unexplored and drug resistance or other selective pressures driving the evolution of these yeasts remained an intriguing question. Comparative genome analysis was carried out to uncover chromosomal aneuploidies and regions with loss of heterozygosity (LOH), two mechanisms that manage genome plasticity. We detected aneuploidy only in human isolates. Bird-derived isolates showed LOH in genes commonly associated with antifungal drug resistance similar to human isolates. Our study suggests that environmental fungicide usage might exert selective pressure on *C. albicans* infecting animals, thus contributing to the spread of potentially resistant strains between different hosts.

## 1 Introduction

Comparative genomics of clinical isolates of yeasts have revealed several genetic events that drive eukaryotic genome dynamics during evolution. *Candida* spp. are capable of rapid and significant genetic changes that may contribute to the successful colonization, persistence, and adaptability across diverse host niches and enhance survival under emerging selective pressures (1). *Candida albicans* has predominantly diploid genome composed by eight heterozygous chromosomes. However, studies with *C. albicans* showed that its genome is shaped by a wide variety of processes including small-scale point mutations, insertions and deletions as well as larger-scale karyotypic rearrangements acting upon ploidy (imbalance in the number of whole chromosomes or chromosomal segments), and zygosity (the number of alleles at a given position in the genome), thus increasing the genomic variations within evolving populations (2, 3).

The genome of *C. albicans* contains a relatively high density of heterozygous positions distributed unequally unevenly in the genome. Rapid adaptation to the environment is also influenced by substantial differences in heterozygosity between isolates. Mitotic recombination between chromosome homologues might result in loss of heterozygosity (LOH) increasing the response to environmental stressors (oxidative stress, high temperature, antifungal drugs) (4). LOH can involve an entire chromosome or partial chromosomal segments due to mitotic crossover or break-induced replication typically extending to the telomeres (5). Although aneuploidy in eukaryotes is commonly accompanied by fitness costs, aneuploid forms of *C. albicans* may confer a selective advantage under certain stress conditions. The reproduction of this yeast is mainly clonal (asexual), nevertheless, parasexual cycle might also occur allowing chromosome shuffling and mitotic recombination events. Mating between diploid strains with opposite mating type locus (*MTLa* and *MTLα*) occurs both *in vitro* and *in vivo*. The result of cell–cell conjugation is a tetraploid form that undergo ploidy reduction via random chromosome loss instead of conventional meiosis to reach diploid or near diploid genomic state (6). These cells are frequently trisomic for one or more whole chromosomes. Harboring supernumerary chromosomes might have a profound effect on the antifungal susceptibility of strains that was evident after isochromosome formation of chromosome 5 yielding resistance to azoles (7).

Although *C. albicans* is a prevalent opportunistic fungal pathogen responsible for superficial and severe systemic infections of humans and animals, most studies investigated clinical isolates from human source and genomic information of isolates from animals are still missing (8). Distinct environmental and host factors might shape the genome structure of *C. albicans* strains in different manner, therefore, comparison of genomes of *C. albicans* isolates originated from different source is essential to better understand the evolution and the adaptation of this species to various host environments. Antimicrobial resistance is an emerging major concern affecting human, animal, and environmental health that prioritize the collective monitoring of infectious diseases and the evaluation of the antifungal susceptibility of pathogenic fungal species from a One Health perspective. Furthermore, the identification of resistance-driving factors including genomic features may help to initiate preventive measures to overcome the spread of resistance (9). The aim of this pilot study was to assess the genomic differences of avian and human *C. albicans* isolates with a special emphasis on antifungal resistance genes and interspecies transmission potential of resistance.

## 2 Materials and methods

### 2.1 Isolates

Species-level identification was carried out with culturing swab samples on Sabouraud dextrose agar supplemented with chloramphenicol, Matrix-assisted laser desorption/ionization time of flight mass spectrometry (MALDI-TOF) and sequencing the internal transcribed spacer (ITS) region of fungal rDNA with universal fungal primers (10). *C. albicans* isolates were collected from humans and birds and the genetic relatedness between strains was investigated by multilocus sequence typing (MLST) method by Domán et al. (11). Thirty *C. albicans* isolates were collected for genotyping. Samples were obtained from ducks and geese diagnosed with oesophageal mycosis (*n* = 22). Isolates from a falcon and an ostrich suffering from gastrointestinal mycosis were also available for genomic analysis. All human isolates (*n* = 6) were cultured from patients with fungal infections (such as decubitus, wound, blood, cervix and pharynx). Out of 30 *C. albicans*, six isolates identified as new genotypes were selected for whole genome sequencing. Human strains were isolated from blood and cervix, while animal-derived isolates originated from esophageal and intestinal samples of birds (Table 1).

**Table 1:** Genomic characteristics of *C. albicans* isolates detected after pair-wise comparison to reference genome SC5314.

Isolate	Host	Sampling site	DST	Number of SNPs	Ploidy	Resistance genes				
						*ERG11*	*ERG24*	*MDR1*	*TAC1*	*FKS1*
14362	Human	Blood	3598	2,937	Diploid	HOM	HET 424 ntp → Y	HET 219 ntp → W 1,651 ntp → R	HOM	HOM
27700	Human	Cervix	3600	3,320	Diploid Chr1, Chr3, Chr5–7, ChrR, triploid Chr2 and Chr4	HET 411 ntp → Y	HOM	HET 1,084 ntp → W	HOM	HOM
Om-8	Duck	Oesophagus	3595	3,727	Diploid	HET 1,470 ntp → Y	HOM	HOM	HET 2,687 ntp → R	HET 939 ntp → Y 2,562 ntp → R
ML-5	Goose	Oesophagus	3599	3,264	Diploid	HOM	HET 1,285 ntp → Y	HOM	HOM	HET 717 ntp → Y 909 ntp → R; 915 ntp → W; 1,350 ntp → Y; 1,359 ntp → Y; 1,653 ntp → Y
Im-12	Ostrich	Intestine	3598	2,970	Diploid	HOM	HET 535 ntp → Y	HOM	HOM	HET 3,239 ntp → Y; 3,264 ntp → W; 3,820 ntp → R
38002	Human	Cervix	3597	1,396	Tetraploid Chr1-3, Chr5–7, ChrR Triploid Chr4	HET 383 ntp → M 658 ntp → Y	HOM	HOM	HET 624 ntp → R	HET 2,079 ntp → R; 2,813 ntp → Y; 3,801 ntp → S

Chr, chromosome; DST, diploid sequence type; HOM, homozygous; HET, heterozygous; ntp, nucleotide position; SNP, single nucleotide polymorphism.

### 2.2 Genome sequencing

Genomic DNA extraction was carried out using the fungi/yeast genomic DNA extraction kit (Favorgen, Taiwan) following the manufacturer’s instructions. Libraries were prepared from genomic DNA using Illumina Nextera XT DNA Library Preparation Kit (Illumina, San Diego, CA, United States) as published elsewhere (12). Whole genome sequencing was performed on Illumina NextSeq 500 sequencing platform (Illumina, San Diego, CA, United States). Single-end reads of 150 nucleotides were generated.

### 2.3 Sequence analysis

**Figure 1:**
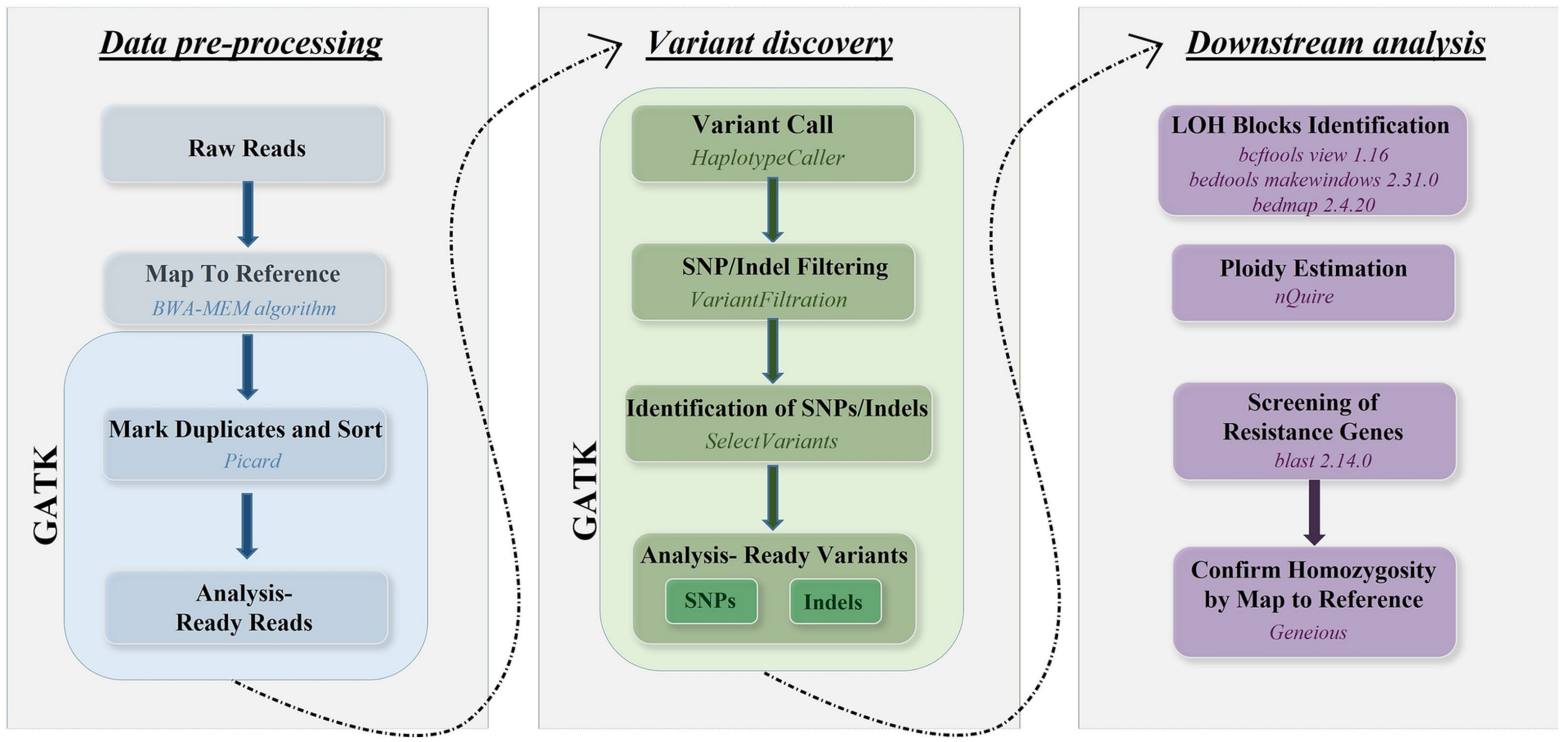
Overview of bioinformatic workflow using reads generated by Illumina platform covering the whole genome of *C. albicans* isolates.

Sequence reads were mapped to the genome of *C. albicans* reference strain SC5314 (Assembly 22) available at Candida Genome Database^1^ using the Burrows–Wheeler Alignment tool (bwa 0.7.17-r1188) with the BWA-MEM algorithm. SNPs were called using the Genome Analysis Toolkit (GATK) v4.2.2.0 (13). Poor quality SNPs and indels were filtered using the GATK VariantFiltration module using the following parameters: QD < 2.0, FS > 60.0, MQ < 40.0, HaplotypeScore > 13.0, MappingQualityRankSum < −12.5, ReadPosRankSum < −8.0. We checked the read depth of SNPs and included only those in the downstream analyses that had a read depth larger than three to decrease the ratio of false positives. Mean sequencing depth and SNPs were illustrated by IGV (2.16.2) (14, 15). The approach validated by Pryszcz et al. (16) was used to define heterozygous and LOH blocks. Briefly, genomic regions having two or more heterozygous sites closer than 100 bases were marked as heterozygous regions. Heterozygous SNPs were filtered using bcftools view 1.16 (17), then the bed files of genomic regions were created using bedtools makewindows 2.31.0 (18). The number of heterozygous SNPs within the bed regions was estimated with bedmap 2.4.20 (19). LOH blocks were considered all non-heterozygous regions in the genome. A 100 bp threshold was established for the minimum LOH and heterozygous block size as well. Additional filtering was used to avoid false positive results according to the following criteria: bases with coverage lower than 5 or higher than 100 were excluded from the analysis. The ploidy of the chromosomes in each sample was estimated separately using nQuire 16.2 (20). After denoising the alignments with nQuire denoise, the ploidy model that had the smallest delta log-likelihood compared to the likelihood of the free model was considered to be supported. SNPs within the resistance genes were identified by alignment of the reference sequences of the resistance genes (Supplementary material) to the reference genome used for the short-read alignments with blastn 2.14.0+ with an expected *e*-value of 1*e*-50 and a similarity cutoff of 95%, then the corresponding genomic regions from the vcf files generated with GATK using bedtools intersect 2.31.0 (18). Homozygosity of resistance genes were confirmed by mapping the reads to both A and B haplotypes of each chromosome of the reference genome in Geneious software (version 2022.2.2). The workflow representing bioinformatic analyses are shown in Figure 1.

## 3 Results

We sequenced six isolates and characterized their variability using 7,527,829 to 9,452,186 short reads. The sequenced isolates were previously assigned as novel MLST genotypes (diploid sequence types, DSTs, https://pubmlst.org/organisms/candida-albicans) (11). Across the 6 isolates, 17,614 SNPs were identified by mapping the reads to the reference genome. The highest number of SNPs were identified in avian isolate Om-8 (*n* = 3,727), whereas the genome of human isolate 38002 contained the least SNPs (*n* = 1,396).

**Figure 2:**
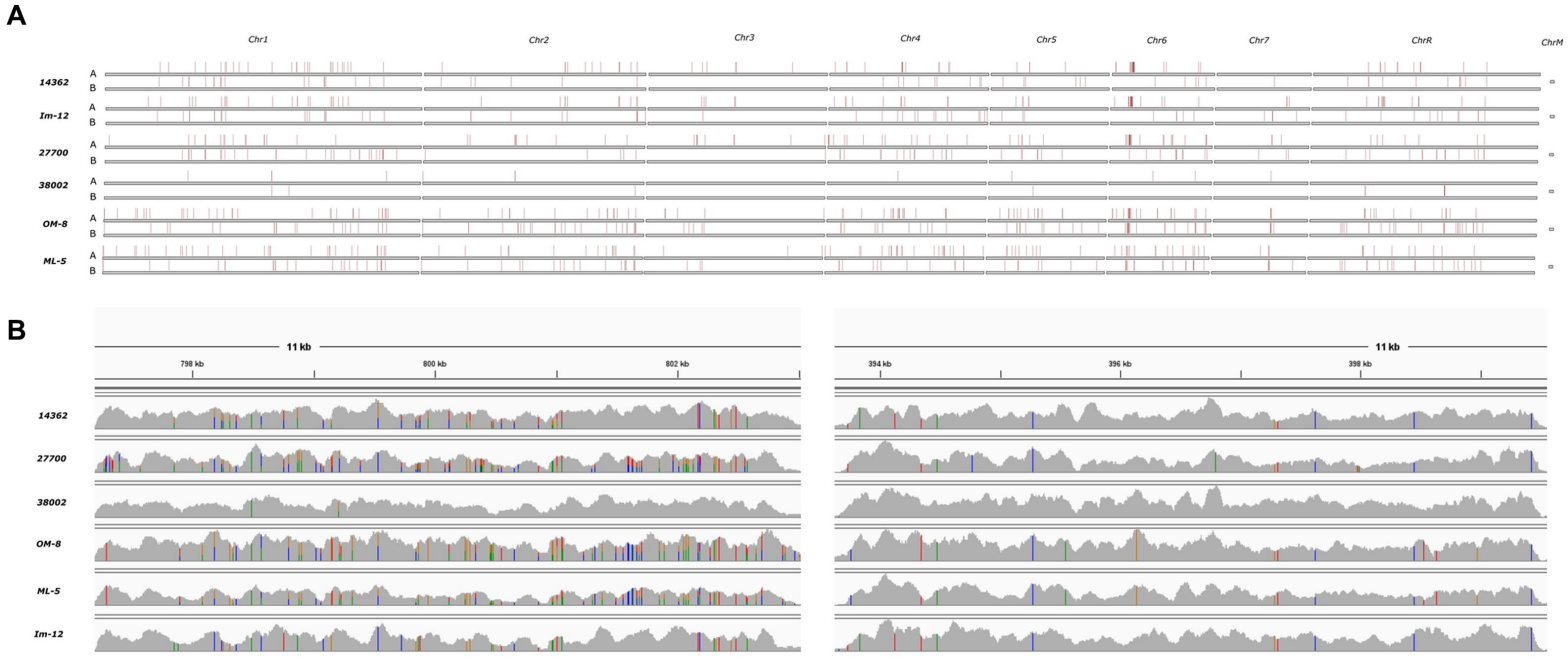
Density of heterozygous SNPs in six sequenced *C. albicans* isolates compared to SC5314 reference strain. **(A)** Heterozygous SNP blocks as defined by Pryszcz et al. (15) (horizontal red stripes) on each chromosomes identified after aligning the short reads to the SC5314 reference genome haplotype A and B. **(B)** Coverage track examples of heterozygous (left) and homozygous (right) genomic regions on chromosome 5 visualized with IGV. Colors are indicative of polymorphic sites. Heterozygous variants are indicated by bars having multiple colors representing the read depth of the given allele.

Chromosomal aneuploidies were observed in only two human isolates. Whole chromosome gains were found in these strains with extra copies of Chr 2 and Chr 4 in isolate 27700. We also observed tetraploidy for nearly all chromosomes in isolate 38002 except for Chr 4 which was triploid. All other isolates appeared to be diploid according to the ploidy models of nQuire. All isolates showed heterozygous mating-type locus; thus, mating-competent isolates were not identified. Heterozygous regions were similarly distributed in the genome of isolates that share the same DST (human 14362 and avian Im-12) or clade (avian Om-8 and ML-5) (11). Overall, homozygous and heterozygous chromosomal regions slightly differed between isolates, however, some genomic trend could be seen in all isolates irrespective of isolation site (e.g., the homozygosity of the right arm of Chr R, large-scale LOH in Chr 3 and Chr7) (Figure 2). SNP analysis revealed that the genome of the human isolate 38002 was the most similar compared to the reference strain SC5314.

We assessed the heterozygosity of genes known to be involved in antifungal drug resistance. Interestingly, LOH was evident in isolates from human source and even in bird-derived isolates as only homozygous regions and a few heterozygous positions were identified in genes responsible for azole resistance (*ERG11*, *ERG24*, *MDR1* and *TAC1*). Four out of 6 isolates contained several heterozygous regions in *FKS1* (certain point mutations in this gene result in echinocandin resistance) and only two human isolates (14362 and 27700) proved to be homozygous for this gene (Table 1). Of note, none of the LOH events were located in hot-spot regions of *FKS1*, in which specific mutations and subsequent amino acid changes are often associated with echinocandin resistance (21).

## 4 Discussion

Fungal strains with stress-induced genomic variations that are potentially advantageous in special circumstances tend to spread and increase the number of these evolved genotypes in the population. Large-scale and rapid genome changes involving whole chromosomes or chromosomal segments occur more frequently than point mutations and have the potential to mediate the response to various stressors (4). In this study, genome-wide analyses were carried out to assess the differences in ploidy, zygosity and gene variations that confer drug resistance between *C. albicans* isolates derived from avian and human hosts. No ploidy shift was detected in strains isolated from birds, but aneuploid state were observed in two out of three human isolates. Notably, both aneuploid isolates originated from cervix indicating that these isolates might exposed to antimycotics and other selective pressures at a higher extent than other sequenced isolates. Several reports are available in the literature discussing that these copy number variations are not always followed by fitness cost and often associated with antifungal drug resistance (22–24). Comparative genome hybridization array showed that 21 out of 42 fluconazole-resistant *C. albicans* isolates carried aneuploid chromosomes (7). In our study, antifungal susceptibility profile was known only in case of human isolates, where fluconazole resistance of isolate 27700 was noticed. This isolate possessed Chr4 trisomy that might contribute to the phenotypic azole resistance (22). While this study shows a high rate of aneuploidy among selected isolates of human *C. albicans* (*n* = 2, 66%), a comprehensive study that analyzed 182 isolates, showed that aneuploidy is a relatively rare phenomenon in *C. albicans* isolates (8).

LOH events with impact on short-or long-range segments of the genome affect fitness under stress conditions providing phenotypic diversity in *C. albicans* isolates. Human infections with *C. albicans* are frequently treated with fluconazole due to its efficacy, low cost, lack of toxicity and ease of administration. Different mechanisms are responsible for azole resistance, such as alterations in the sterol biosynthesis pathway, increased expression of the *ERG11* gene encoding the drug target enzyme, mutations in Erg11p that result in reduced binding capacity of fluconazole to its target protein, and reduced effective drug concentration in the cells by overexpression of multi-drug efflux pumps. Elevated drug resistance might occur via mutations of *ERG11*, *TAC1* or *MRR1* followed by LOH that alter azole drug targets or increase drug efflux (25). Interestingly, there were no significant differences between *C. albicans* isolates originated from different host species regarding LOH of antifungal drug resistance genes. Most of the isolates were homozygous for genes associated with azole resistance in contrast with reference strain SC5314. As isolates Om-8 and ML-5 were obtained from fattened goose and duck which were not treated with any antifungal drugs due to food safety issues, perhaps another selective pressure or route of drug exposure result in LOH in these genes. This finding raises the possibility that repeated exposure to agricultural fungicides that are structurally related to fluconazole accumulated in water, soil, or in the food chain might exert selective pressure on *C. albicans* isolates colonizing or infecting animals (26). A recent study has also reported fluconazole-resistant *C. albicans* from chicken crop mycoses and calf diarrhea (27). Animals then may shed these *C. albicans* strains with relevant genetic variations to the environment and enable intra-or interhost transmission of yeast resistant to various antimycotics in the absence of previously documented drug exposure.

Nowadays, the management of health policies in a One Health perspective is essential to control the spread of diseases. New technologies like geospatial analysis tools might reveal environmental patterns that facilitate disease transmission between animals and humans. New methodologies like geographic information system (GIS) and remote sensing tools largely contributed to better understanding of disease dynamics as associations between environmental conditions and infectious diseases were recognized (28, 29). Genome-based approaches coupled with GIS may serve as a pioneering method in veterinary and human medicine to prevent the spread of diseases in ecosystems. Investigations using larger dataset and these new methods might identify risk factors of *C. albicans* transmission between individuals and populations (e.g., associations between genotypes and environmental factors) underlining the importance of metadata and surveillance studies.

## 5 Conclusion

Only a few studies are available in the literature that examine antifungal drug resistance of animal-derived *C. albicans* isolates. Moreover, information on genomic changes that might associated with resistance in *C. albicans* originated from animals is still lacking. Although our study has some limitations (including the low sample size, the lack of confirmation of genome sequencing data by susceptibility testing methods), we showed here by whole genome sequencing method that poultry might serve as source of *C. albicans* strains resistant to antifungal drugs commonly used in human medicine. This finding highlights the importance of One Health approach to prevent the spread of drug resistant fungal species and justifies the extension of comparative genomics on animal origin *C. albicans*. Increased surveillance of antifungal resistance within animals and the environment could provide significant information to integrate control measures for disease prevention even in the absence of fungicides.

## Data availability statement

The datasets presented in this study can be found in online repositories. The names of the repository/repositories and accession number(s) can be found at: https://www.ncbi.nlm.nih.gov/, PRJNA1020061.

## Author contributions

MD: Conceptualization, Data curation, Formal analysis, Funding acquisition, Methodology, Writing – original draft. EK: Formal analysis, Software, Writing – review & editing. LL: Formal analysis, Software, Writing – review & editing. KBa: Formal analysis, Methodology, Writing – review & editing. LMak: Methodology, Resources, Writing – review & editing. RK: Methodology, Resources, Writing – review & editing. LMaj: Resources, Supervision, Writing – review & editing. KBá: Conceptualization, Funding acquisition, Supervision, Writing – review & editing.

## Footnotes

^1^ http://www.candidagenome.org/download/sequence/C_albicans_SC5314/
