# Supplementary material for: Genomic epidemiology of antifungal resistance in human and avian isolates of Candida albicans: a pilot study from the One Health perspective
Source: Front Vet Sci. 2024 Feb 16;11:1345877. doi: 10.3389/fvets.2024.1345877 (PMC10904516; doi:10.3389/fvets.2024.1345877)
Supplement: Supplementary file 1 [file Data_Sheet_1.docx]

Supplementary Material

**Supplementary table 1.** Data on genes associated with antifungal drug resistance used for genome-wide analysis.

| **Genes** | **Protein/function** | **Mechanism of resistance** | **Chromosome** | **Reference sequences of chromosomes in GenBank** | **Location of genes in the chromosome** | **Length (bp)** |
| --- | --- | --- | --- | --- | --- | --- |
| *ERG1* | squalene epoxidase | point mutation | 1 | NC_032089.1 | 1,882,037-1,880,547 | 1,491 |
| *ERG2* | C-8 sterol isomerase | point mutation, insertion | 1 | NC_032089.1 | 155,286-154,633 | 654 |
| *ERG3* | C-5 sterol desaturase | point mutation | 1 | NC_032089.1 | 992,782-991,622 | 1,161 |
| *ERG5* | putative C-22 sterol desaturase | point mutation | 7 | NC_032095.1 | 613,691-612,138 | 1,554 |
| *ERG6* | delta(24)-sterol C-methyltransferase | point mutation | 3 | NC_032091.1 | 466,125-464,995 | 1,131 |
| *ERG11* | lanosterol 14-alpha-demethylase | overexpression/point mutation | 5 | NC_032093.1 | 149,701-148,115 | 1,587 |
| *ERG24* | C-14 sterol reductase | point mutation | 2 | NC_032090.1 | 1,914,592-1,913,246 | 1,347 |
| *ERG251* | C-4 sterol methyl oxidase | point mutation | 4 | NC_032092.1 | 318,105-317,140 | 966 |
| *UPC2* | Zn2-Cys6 transcript factor | point mutation | 1 | NC_032089.1 | 1,860,274-1,858,136 | 2,139 |
| *CDR1* | multidrug transporter of ABC superfamily | overexpression | 3 | NC_032091.1 | 1,146,045-1,150,550 | 4,506 |
| *CDR2* | multidrug transporter of ABC superfamily | overexpression | 3 | NC_032091.1 | 1,047,887-1,052,386 | 4500 |
| *MDR1* | plasma membrane MDR/MFS multidrug efflux pump | overexpression | 6 | NC_032094.1 | 668,077-666,383 | 1,695 |
| *TAC1* | Zn(2)-Cys(6) transcriptional activator of drug-responsive genes (CDR1 and CDR2) | point mutation | 5 | NC_032093.1 | 419,345-416,400 | 2,946 |
| *MRR1* | putative Zn(II)2Cys6 transcription factor; regulator of MDR1 transcription | point mutation | 3 | NC_032091.1 | 1,329,263-1,332,598 | 3,327 |
| *MRR2* | Zn(II)2Cys6 transcription factor involved in regulation of multidrug resistance via control of CDR1 expression | point mutation | 3 | NC_032091.1 | 1,780,547-1,778,415 | 2,133 |
| *FKS1* | beta-1,3-glucan synthase subunit | point mutation | 1 | NC_032089.1 | 1,169,206-1,173,921 | 4,716 |
| *FKS2* | beta-1,3-glucan synthase subunit | point mutation | RA | NC_032096.1 | 197,094-192,172 | 4,923 |
| *GLS1* | alpha glucosidase I | point mutation | 4 | NC_032092.1 | 1,355,313-1,357,805 | 2,493 |


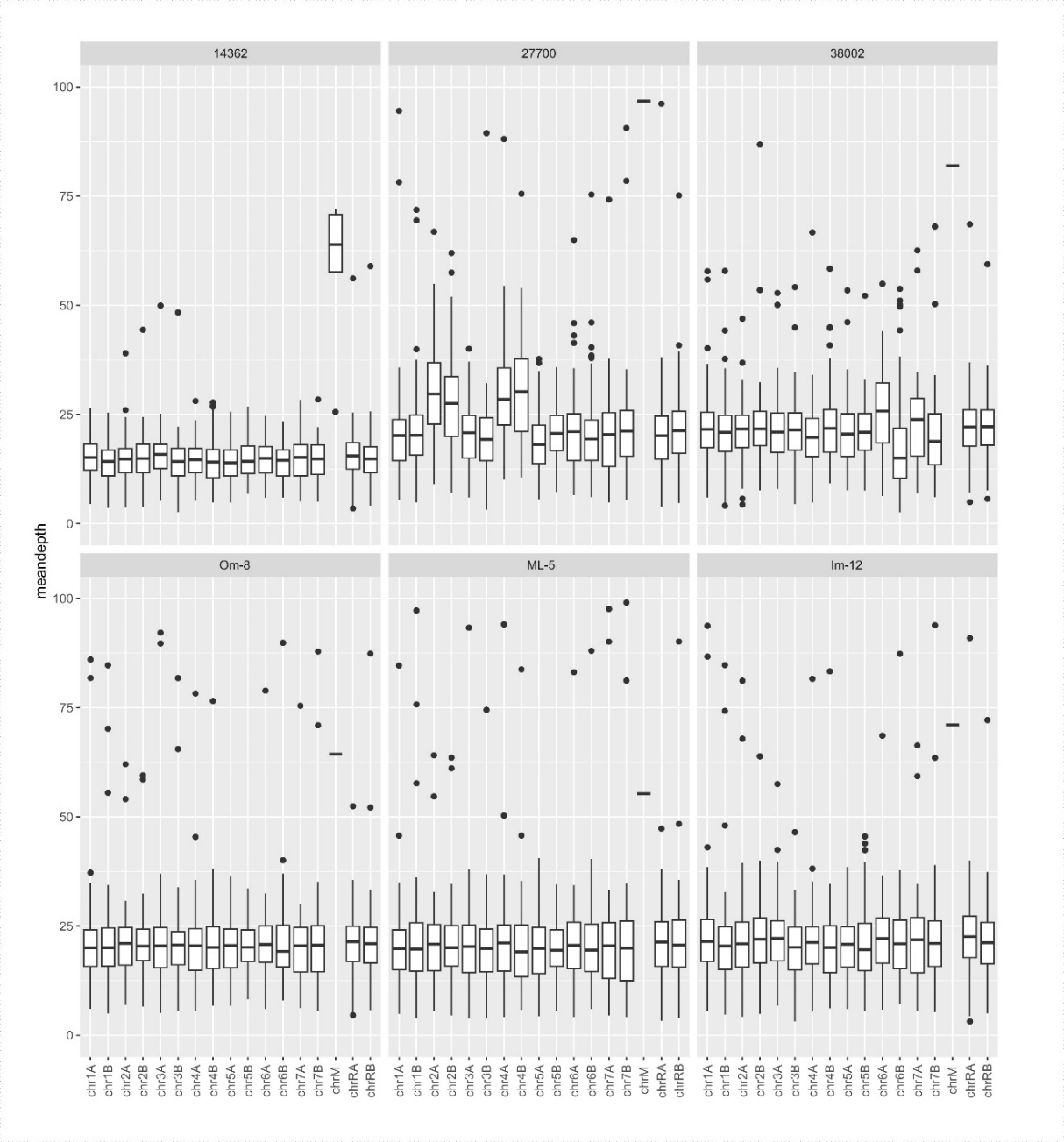


**Supplementary Figure 1.** Sequencing depths of C. albicans isolates for each chromosomal haplotype
